# Treatment of Patients with Monoclonal Gammopathy of Clinical Significance

**DOI:** 10.3390/cancers13205131

**Published:** 2021-10-13

**Authors:** David F. Moreno, Laura Rosiñol, María Teresa Cibeira, Joan Bladé, Carlos Fernández de Larrea

**Affiliations:** 1 Amyloidosis and Multiple Myeloma Unit, Hospital Clínic, 08036 Barcelona, Spain; dfmoreno@clinic.cat (D.F.M.); lrosinol@clinic.cat (L.R.); mcibeira@clinic.cat (M.T.C.); jblade@clinic.cat (J.B.); 2 Institut d’Investigacions Biomèdiques August Pi i Sunyer (IDIBAPS), 08036 Barcelona, Spain

**Keywords:** MGCS, MGUS, skin, ocular, bleeding

## Abstract

**Simple Summary:**

Monoclonal gammopathy of clinical significance (MGCS) is a recently recognized clinical-pathological entity. Symptoms are caused by the presence of a monoclonal protein leading to high comorbidity. The affected organs vary according to the target antigen However, as most of the knowledge relies on case reports or short series; there is a lack of consensus regarding treatment approach. Here, we discuss MGCS other than renal (skin, ocular, neurologic, and bleeding disorders). We provide insights into the pathophysiology, diagnosis, treatment, and follow-up based on clinical cases. Finally, we discuss future directions in this field, such as potential novel therapeutic targets and prognosis of patients with MGCS.

**Abstract:**

Monoclonal gammopathy of undetermined significance (MGUS) is defined as the presence of a monoclonal protein (M-protein) produced by a small amount of plasma cells. The majority of patients remain asymptomatic; however, a fraction of them develop clinical manifestations related to the monoclonal gammopathy despite not fulfilling criteria of multiple myeloma or other lymphoproliferative disorder. These patients constitute an emerging clinical issue coined as monoclonal gammopathy of clinical significance (MGCS). The mechanisms involved are poorly understood, and literature is scarce regarding management. The clinical spectrum involves symptoms related to renal, neurologic, skin, ocular, or bleeding manifestations, requiring a multidisciplinary approach. Treatment strategies rely on the basis of symptomatic disease and the M-protein isotype. In this review, we focus on MGCS other than renal, as the latter was earliest recognized and better known. We review the literature and discuss management from diagnosis to treatment based on illustrative cases from daily practice.

## 1. Introduction

Monoclonal gammopathy of undetermined significance (MGUS) is defined by the presence of a monoclonal protein (M-protein) produced by a small B-cell/plasma cell clone in persons without features of symptomatic disease related to malignant disorders, such as multiple myeloma (MM), Waldenström macroglobulinemia (WM), AL amyloidosis, or other lymphoproliferative disorder [1,2]. Prevalence is around 3% among people older than 50 years, and it increases with age [3]. Nearly 80% of MGUS cases are derived from a non-IgM isotype (IgG or IgA), with IgG the most frequently found in population-based studies [4]. In the absence of myeloma-related symptoms, non-IgM MGUS is characterized by an M-protein lower than 30 g/L and less than 10% of plasma cells in bone marrow. Similarly, light-chain MGUS is based on an increased concentration of the involved light chain rather than a heavy-chain immunoglobulin expression, causing an abnormal free light chain ratio [2]. In the absence of WM-related symptoms, IgM MGUS is defined by an M-protein lower than 30 g/L and less than 10% of lymphoplasmacytic cells in the bone marrow [5,6]. Risk of progression to a malignant disorder (MM, WM, AL amyloidosis, or other B-cell lymphoproliferative disorder) is estimated in 1% annually [4,7]. This shortens the overall survival (OS) for those who have MGUS compared to the control-matched population [4].

Despite the above mentioned, the majority of these patients will not progress to overt MM or other lymphoproliferative neoplasm and die from unrelated disorders. Apart from the potential malignant evolution, in some instances, the presence of a small B-cell/plasma cell clone producing a monoclonal immunoglobulin may cause a wide variety of clinical manifestations, leading to a significant comorbidity and need for treatment [8,9]. Given the high heterogeneity of organs involved and clinical manifestations, this spectrum is categorized by an emergent concept recently coined as monoclonal gammopathy of clinical significance (MGCS) [10].

In many instances, the mechanisms underlying MGCS are unclear. Given the diversity of clinical manifestations, they are divided by organ involvement to better understand the pathophysiology, diagnosis, and treatment. In this sense and because of the high comorbidity and relative frequency, the most affected and well-studied organ is the kidney. So far, kidney disease related to the presence of an M-protein was already categorized as monoclonal gammopathy of renal significance (MGRS) [11,12,13].

Other MGCS are those involving the eyes, skin, peripheral nerves, and coagulation system, among others. Clinical manifestations may overlap with other unrelated M-protein diseases, making them hard to diagnose and to make related treatment decisions [14]. As MGRS, the aim of this review is to discuss MGCS other than renal, more recently recognized, highlighting practical diagnostic aspects and treatment approach.

## 2. Pathophysiology of MGCS

The mechanisms by which the presence of the M-protein results in clinical syndromes not related to progression to a plasma cell or lymphoproliferative neoplasms can be divided according to findings on histopathology. Among these, the M-protein deposition is a common feature of many MGCS. As suggested by Fermand et al., the abnormal biochemical conformation of the monoclonal immunoglobulin results in its deposition that can be classified whether they have organized or non-organized ultrastructural appearance [10]. For instance, crystalline deposits of monoclonal immunoglobulin in the cornea are the main cause of keratopathy [15]. In the case of type 1 cryoglobulinemia, intravascular monoclonal deposits (IgM or IgG) are microtubular or crystalline, causing thrombotic occlusion of small vessels under the skin and, with less frequency, in the kidney or peripheral nerves [9,16]. Another example is crystal-storing histiocytosis, a rare disease associated with an underlying monoclonal gammopathy that has intralysosomal M-protein deposits with crystal composition [17]. Although not part of this review, it is important to point out that AL amyloidosis is by far the most characterized disease concerning the nature of fibrillar deposits [18]. On the other hand, non-organized deposits are common features of monoclonal immunoglobulin deposition disease, causing renal damage in the majority of cases [19].

The monoclonal immunoglobulin may also interact with several self-antigens, causing disease. The autoantibody effect of the M-protein can facilitate an autoimmune response depending on the target self-antigen. This process is seen in IgM-peripheral neuropathy, as the IgM binds directly to gangliosides or myelin glycoproteins (MAG). A relevant epitope in anti-MAG neuropathy is the HNK-1 (human natural killer-1) that is located in the peripheral nervous system. The presence of an autoantibody blocks the physiologic signaling and regulatory processes of MAG resulting in the clinical manifestations [20,21,22]. In the case of bleeding disorders related to the M-protein, it is reported that the monoclonal immunoglobulin increased the degradation of von Willebrand factor (VWF) [23]. Platelet dysfunction has also been described when the M-protein deposits to surface antigens, such as GP-1b (glycoprotein-1b) or GP-IIIa [24]. However, it remains unclear the high affinity of certain M-proteins to bind these specific antigens. On the other hand, the mere presence of the plasma cell clone can induce abnormal secretion of EGF (epidermal growth factor) and MCP-1 (monocyte chemoattractant protein-1), or the interaction between monoclonal IgA with its receptors can also induce release of pro-inflammatory mediators [25]. Both approaches explain the underlying mechanism in pyoderma gangrenosum associated with IgA M-protein.

Detecting molecular patterns of disease using high-throughput technologies may raise more solid basis on understanding better MGCS as a different clinical-pathological entity. For instance, sequencing Schnitzler syndrome has revealed a unique upregulation of the inflammasome pathway [26]. In the case of scleromyxedema, transcriptomic analysis of the skin revealed high expression of TGF-β (transforming growth factor-β) [27]. Furthermore, the B-cell molecular status in anti-MAG neuropathy has given some understanding regarding its clonal origin. In fact, *MYD88^L265P^/CXCR4^wt^* and the identification of the *VH4-34* segment in the *IGH* loci were more prevalent when compared to IgM MGUS and WM, giving more insight in the clonal origin of the disease [28]. Besides all of these, the question to be solved is why some MGUS patients develop clinical symptoms related to the M-protein and the vast majority not. The ability of the monoclonal immunoglobulin to cause a clinical significance in MGUS still remains unknown. So far, neither the amount of the M-protein nor malignant clones are the answers. Testing the malignant clone with its immune microenvironment in addition to the affected tissue may answer this question. 

From the clinical perspective, MGCS can be categorized regarding the involved organ. This practical approach resembles what is seen at the clinic. Although some of them share the same involved organs (i.e., type 1 cryoglobulinemia has multisystemic involvement), the MGCS list includes the most important diseases with the cardinal involved organ (i.e., skin for type 1 cryoglobulinemia) (Table 1).

## 3. Skin Disorders

### 3.1. Type 1 Cryoglobulinemia

Cryoglobulinemia can damage any organ, but the skin is usually the most frequent location. Type 1 cryoglobulinemia is caused by plasma cell or lymphoproliferative disorders, and it is mainly due to IgM or IgG M-protein [16]. Clinical manifestations are related to a vasculitis, resulting in petechiae, purpura, and ulcers. Some of these lesions can be cold-induced, with repeated episodes of livedo and purpura (vasomotor symptoms). Sensory peripheral neuropathy is the second system affected [9]. Glomerulonephritis is rare and is caused by small-vessel occlusion due to intravascular deposition [12].

Treatment depends on the severity of symptoms and the underlaying cause. Besides WM-associated cryoglobulinemia that has international consensus [29], there is no current standard recommendations for treatment. The first step is to explain and educate patients that cold exposure can exacerbate vasomotor symptoms. Wearing warm clothes to protect hands and feet when exposed to cold temperature is necessary [30]. However, patients with overt skin lesions are usually seen. In this scenario, the next step should be focused on the underlying disease. Single-agent prednisone may control the disease in patients with low tumor burden (IgG or IgM MGUS) [30]. In the case of WM, the initial approach should be the current recommended therapy for this disease [29,30,31].

In patients with MM, combination of proteasome inhibitors and immunomodulatory drugs can achieve good responses before autologous stem cell transplant (ASCT). In a report of 46 patients with an underlying IgG M-protein, most of them responded well to the cryoglobulinemia symptoms whether using bortezomib, alkylating agents, immunomodulatory drugs, or high-dose melphalan. With these data, type 1 cryoglobulinemia patients had 5- and 10-year estimated survival rates of 83 and 68%, respectively [16].

**Figure 1:**
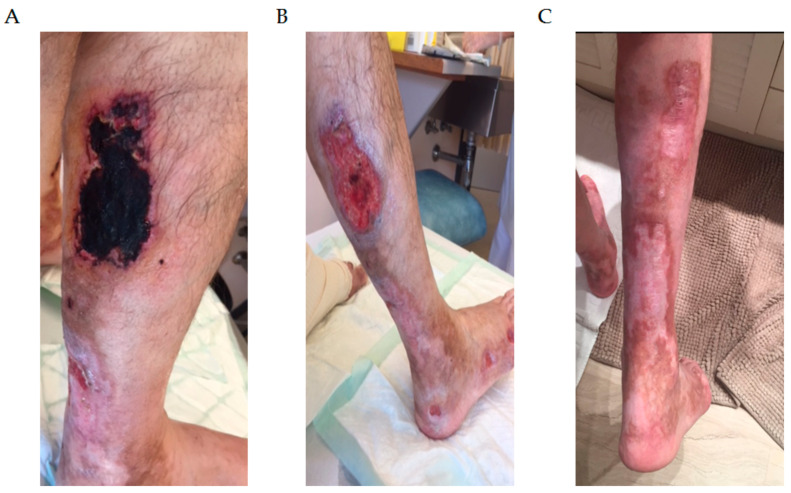
Patient diagnosed with multiple myeloma and type 1 cryoglobulinemia, causing severe skin ulceration on lower extremities. (**A**) Skin ulcers before daratumumab. (**B**) Improvement of the skin condition during treatment. (**C**) Complete resolution of the skin ulcers with daratumumab.

Clinical case 1: A 63-year-old male was admitted because of a 12-month history of skin lesions in the legs and both feet. At that time, blood and basic biochemistry lab tests did not show any abnormality. Autoimmunity and viral serologies in serum were all negative. He was prescribed oral antibiotics because of the suspicion of an infectious disease. However, the skin lesions progressed to painful ulcers and extension to both feet. The skin biopsy showed thrombosis in small vessels. Given a suspicion of an autoimmune disorder, the patient was started on oral corticosteroids with no improvement. Because of the progression of the skin lesions, the patient was referred to a tertiary hospital, where screening tests showed a biclonal M-protein (IgG-kappa and IgA-lambda) by serum immunofixation. Serum cryoglobulins were positive for type 1 cryoglobulinemia. The bone marrow aspirate showed 2% of plasma cell infiltration by optical microscopy morphology (only 30% of them had abnormal immunophenotype), and whole-body CT scan showed osteolytic lesions in right humerus and the skull. In this scenario, the patient was diagnosed with type 1 cryoglobulinemia related to MM and started induction treatment with bortezomib, thalidomide, and dexamethasone followed by ASCT, achieving hematologic complete remission. The skin lesions improved substantially through each cycle of treatment until complete resolution. Two years later, he relapsed in form of symptomatic cryoglobulinemia and bone lesions. He was started on lenalidomide and dexamethasone with no response. Then, ixazomib, lenalidomide, and dexamethasone were considered, but the skin condition did not respond. The third line of treatment was pomalidomide and dexamethasone, but progression was otherwise seen, and the skin ulcers on the leg were severely affected (Figure 1A). The following treatment was single-agent daratumumab, achieving hematological partial response with resolution of the skin condition. Remission of the skin lesions was seen during each cycle (Figure 1B,C). One and a half years later, the patient developed an abrupt serological and clinical myeloma progression with no reappearance of the skin lesions. He was included in a clinical trial using anti-BCMA antibody-drug conjugate [32]. After two cycles showing stable disease, he suffered a severe bacterial pneumonia and passed away.

### 3.2. Schnitzler Syndrome

Schnitzler syndrome is an autoinflammatory disease with an IgM M-protein (rarely IgG) that presents in form of chronic urticaria. According to Strasbourg criteria, major criteria include chronic urticaria rash and IgM or IgG M-protein. Minor criteria are recurrent fever, leukocytosis and/or elevated C-reactive protein (CRP), neutrophilic dermal infiltrate on skin biopsy, and abnormal bone remodeling that may lead to bone pain or arthralgias [33]. To diagnose Schnitzler syndrome, patients need to have both major criteria and two minor criteria if IgM M-protein is present or three minor criteria in the case of IgG M-protein. Probable Schnitzler syndrome includes the presence of both major criteria and one or two minor criteria for each isotype, respectively [33,34]. Given the inflammatory background of the disease, antagonizing interleukin 1 (IL1) with anakinra achieves good control of disease and long remission [26,33,35]. Anakinra is started at 100 mg/d subcutaneously until symptoms are controlled. Then, it could be tapered at the lowest possible dose until resolution of skin lesions. In a study of 21 patients with Schnitzler syndrome treated with anti-IL1, 95% of them achieved clinical remission. Moreover, with a median follow-up of 64 months, none of them required chemotherapy [26]. Colchicine and steroids are also acceptable options, especially when tapering anakinra up to complete stop (flares can appear after anakinra interruption) [33]. New anti-IL1 rilonacept and canakinumab can be also considered [33,36]. However, some patients may relapse after long-term remission or do not tolerate chronic therapy with the options above mentioned. As other MGCS, therapy against the underlying disease should also be considered in case of refractory disease impairing quality of life. Although there are few reports in refractory disease, it is described that treatment based on anti-CD20 can control symptoms in IgM-related disease [36]. There are no case reports or studies that demonstrate effectiveness of anti-myeloma agents in case of non-IgM Schnitzler syndrome. In our experience, it could be reserved only for patients who are severely affected by the disease and for whom no response is achieved with the above mentioned treatments. Here, we present two cases that illustrate a typical IgM Schnitzler syndrome and an unusual non-IgM type who is, by our knowledge, the first patient that was treated with ASCT.

**Figure 2:**
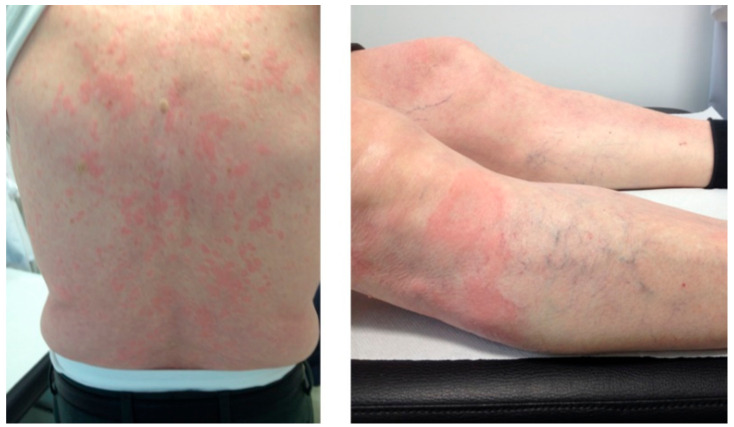
A 78-year-old patient with urticarial lesions, fever, mild leukocytosis, elevated CRP, and positive immunofixation for IgM kappa. The patient was diagnosed with Schnitzler syndrome.

Clinical case 2: A 78-year-old male was referred to our center because of repeated episodes of urticaria on the trunk in the last year. He also complained of fever and arthralgia in each episode. At physical examination, he had pruriginous skin papules on the back and legs (Figure 2). Lab tests showed mild leukocytosis (14,000 cells/μL), positive serum immunofixation for monoclonal IgM-kappa, and elevated CRP. The M-protein was 1.3 g/L. Skin biopsy showed dermal infiltration by neutrophils and lymphocytes without vasculitis. The bone marrow aspirate had 12% of lymphocytes by morphology. However, flow cytometry analysis showed a normal distribution of B cells by immunophenotype (1% out of the total cellularity) without any clonal population. *MYD88* L265P mutation was positive by allele-specific polymerase chain reaction (AS-PCR). The patient was diagnosed with Schnitzler syndrome related to IgM MGUS and was started on 0.5 mg/kg/d oral prednisone. After 2 months of treatment and tapering the prednisone dose, the patient achieved good response of the skin lesions. Six years later, the patient is still on low-dose prednisone (7.5 mg daily) because when discontinued, he complained again of mild fever and urticaria. During the 6-year follow-up, the M-protein has had an evolving pattern up to 12 g/L, but there are no signs of progression to a lymphoproliferative disorder or further extra-hematological activity.

Clinical case 3: A 43-year-old female was referred to our center because of chronic urticaria, fever, and arthralgia not responding to symptomatic treatment. Lab tests showed mild leukocytosis (14,250 cells/μL) and positive serum immunofixation for monoclonal IgG-kappa. Serum electrophoresis showed 10.7 g/L of M-protein. The bone marrow contained 4% plasma cells, most of them with abnormal immunophenotype. Skeletal survey did not show bone lytic lesions. She was diagnosed with probable Schnitzler syndrome related to an IgG-kappa monoclonal gammopathy and started treatment with oral corticosteroids and methotrexate without response. Hydroxychloroquine and anakinra were also tried, but the patient did not show any improvement after two months and developed intolerance to anakinra. In this scenario, treatment against the plasma cell clone with bortezomib was prescribed. After three cycles, she achieved complete response of the skin lesions and a serological very good partial response (VGPR) of the monoclonal gammopathy. She underwent ASCT and achieved complete serological response. After 6 years, she is still in complete remission without recurrence of the Schnitzler syndrome.

### 3.3. Pyoderma Gangrenosum

This entity consists of neutrophilic dermatoses that cause an ulcerative skin condition. It usually starts as a single or multiple ulcerative nodules with violaceous borders in any area of the body [37]. Most cases are associated with systemic inflammatory diseases; however, recent reports suggest MGUS as an underlying cause [38]. IgA isotype has been described as the more prevalent, followed by IgM [25]. Treatment depends on the severity of the skin condition and the underlying cause. Topical or oral corticosteroids may be an initial approach. Tumor necrosis factor inhibitors, such as infliximab, have been studied in other inflammatory diseases with good response and may be an option to consider. Other steroid-sparing drugs, such as oral dapsone, cyclosporine A, mycophenolate, and tacrolimus, have been tried [37,39].

**Figure 3:**
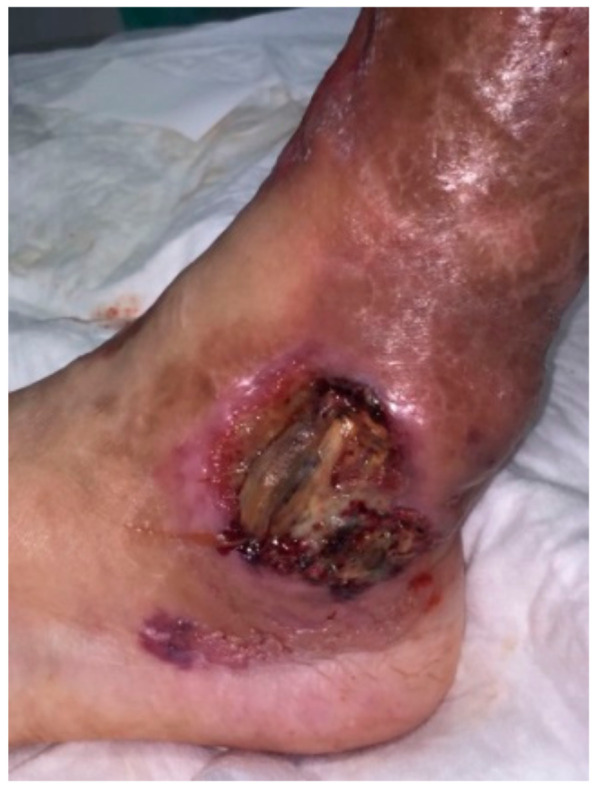
Skin ulcer on left ankle with violaceous elevated borders in a patient with a long history of pyoderma gangrenosum and IgA-kappa monoclonal gammopathy of undetermined significance.

Clinical case 4: A 60-year-old male had been suffering from episodic, painful skin ulcers on both legs after minimal accidental trauma. A skin biopsy was consistent with pyoderma gangrenosum. Serum immunofixation and electrophoresis showed a monoclonal IgA-kappa of 8 g/L without any other myeloma-related features (bone marrow aspirate had less than 10% plasma cells with normal immunophenotype, and skeletal survey did not show bone lytic lesions). The screening for systemic inflammatory diseases was also negative. After an initial approach with oral corticosteroids, the patient did not respond. Cyclosporin, tacrolimus, and mycophenolate were tried without disease control and developing renal toxicity. Finally, infliximab achieved good response of the ulcers. After 6 years of treatment for the pyoderma gangrenosum, the patient relapsed with ulcers on the left ankle (Figure 3) and complained of back pain. The MRI showed pathologic vertebrae fractures on T7 and T12 and a soft-tissue mass. The biopsy showed aberrant plasma cell infiltration compatible with a paraskeletal plasmacytoma. Progression to symptomatic MM was diagnosed, and he started induction treatment. However, he died because of severe coronavirus disease 2019 (COVID-19) pneumonia.

### 3.4. Scleromyxedema

Scleromyxedema is a generalized sclerodermoid form of lichen myxedematosus, also known as a type of dermato-neuro syndrome (DNS), typically associated with the presence of an IgG M-protein. Systemic involvement includes cardiomyopathy, pulmonary fibrosis, joint contractures, and reduced esophageal motility [40]. Transcriptomic analysis has shown increased autoinflammatory pathway activation (collagen and mucin production) [27]. Treatment options include intravenous immunoglobulin (IVIG) as first line, while anti-myeloma agents are considered as second options [40]. Recently, it has been suggested that lenalidomide or bortezomib plus dexamethasone combined with maintenance infusions of IVIG are promising options with good clinical responses in relapsed/refractory scleromyxedema. A recent report on 33 patients using plasma exchange (PE) and anti-myeloma agents showed a 97% survival rate at 3 years [27].

**Figure 4:**
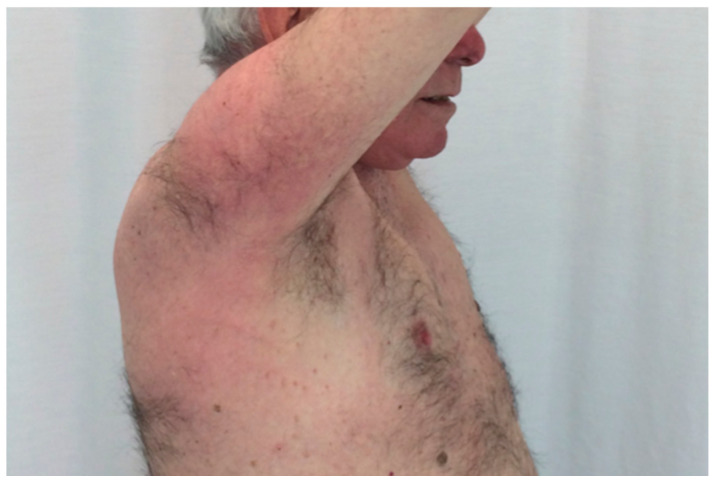
Rigid sclerodermoid lesions on right arm and shoulder in a patient with IgG kappa monoclonal gammopathy.

Clinical case 5: An 80-year-old male was referred because of sclerodermoid lesions on the upper and lower extremities causing rigidity limiting physical activity (Figure 4), dysphagia, weight-loss, and the presence of an IgG-kappa by serum immunofixation. Autoimmunity tests related to systemic sclerosis and CREST syndrome were all negative. Skin biopsy showed increased collagen fibers in dermis consistent with a sclerodermoid form. The M-protein was 17 g/L and 7% of plasma cells were observed in the bone marrow aspirate (97% with abnormal immunophenotype). No lytic lesions were found on skeletal survey, and no other myeloma-related features were found in the screening tests. In this scenario, the patient was diagnosed with scleromyxedema associated to IgG-kappa MGCS. Given the important comorbidity that the disease was causing, treatment with melphalan, prednisone, and bortezomib was administered. After five cycles, the patient substantially improved, and it was decided to keep under observation. During the next 6 years of follow up, the patient has not required further therapy against the plasma cell clone, with stable serum M-protein.

### 3.5. Acquired Generalized Cutis Laxa

Acquired cutis laxa is a rare skin condition that is associated with prior inflammatory diseases that results in elastolysis [41,42]. However, recent reports showed that the presence of an underlying monoclonal gammopathy as a potential cause [43,44,45]. In a series of 42 patients with cutis laxa and monoclonal gammopathies, IgG isotype was the most prevalent [44]. Cutis laxa is characterized by inelastic and pendulous skin, especially in the axilla, groin, and neck. Because of the elastolysis of the skin, patients usually have the appearance of “premature aging”. Rarely, extra-cutaneous manifestations include pulmonary, gastrointestinal, genitourinary, and cardiovascular involvement [43,46]. Treatment is directed to the underlying gammopathy.

**Figure 5:**
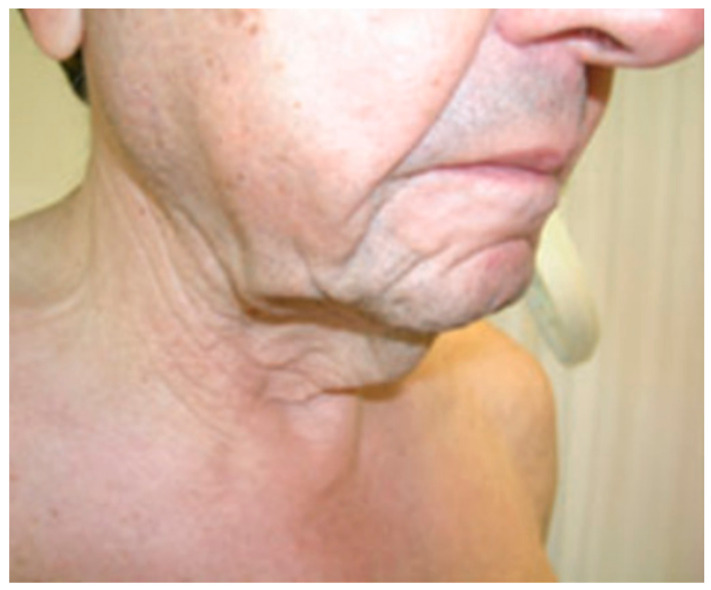
A 52-year-old patient that complaint of premature aging. Skin looks inelastic and pendulous on the neck. Immunofixation was positive for IgG-lambda. Skin biopsy was consistent with cutis laxa.

Clinical case 6: A 52-year-old male was referred because of progressive skin changes in the last 2 years in the form of inelastic skin on body fold areas (face, neck, axillae, and groins—Figure 5). Symptoms worsened during the last 3 months, with addition of bilateral malleolar edema and fatigue. Lab tests showed mild anemia (110 g/L) and high serum creatinine level (2.7 mg/dL). Serum electrophoresis and immunofixation demonstrated an IgG-lambda M-protein of 4.4 g/L. The 24-hour urine protein excretion was 2.7 g (glomerular non-selective pattern). The bone marrow aspirate showed 5% of plasma cells, and skeletal survey was normal. In this context, it was considered to perform skin and kidney biopsies. The skin histopathology showed a reduction of elastic fibers in the dermis and even absence in some areas. Immunofluorescence was positive for IgG deposition in the dermoepidermal junction and periadnexial areas. The kidney biopsy showed fibrillar glomerulonephritis, negative for Congo red staining. Otherwise, pulmonary functional tests, CT body scan, and echocardiography did not show any other abnormalities. He was diagnosed with generalized acquired cutis laxa with nephrotic syndrome associated to IgG-lambda MGCS. The patient was considered fit for ASCT; however, he suffered from alveolar hemorrhage and acute kidney injury during the stem cell mobilization leading to hemodialysis. For the MGCS, he was started on bortezomib and oral dexamethasone for six cycles and achieved complete hematological response. The skin condition was stable, and surgical correction was performed. Three years later, he underwent a kidney transplant without any complications. After eight years of clinical and serological response, the IgG-lambda M-protein reappeared. He was started again on bortezomib and dexamethasone therapy for six cycles and achieved a second complete response with no relapse so far. Thus, the patient has completed now 14 years of follow-up since diagnosis.

*Treatment summary recommendation of skin related MGCS.* Type 1 cryoglobulinemia responds to corticosteroids, cyclophosphamide, and PE in the absence of overt malignancy. If the underlying M-protein is IgM, rituximab and/or alkylating agents may be considered. Severe cases or the presence of underlying MM may respond to anti-myeloma agents. Schnitzler syndrome treatment is based on anti-IL1 agents (anakinra), with effective remission of symptoms. Anti-myeloma agents should be used only in refractory disease. Non-severe scleromyxedema treatment with IVIG can be considered. For refractory or severe manifestations, addition with anti-myeloma agents can achieve hematological and clinical response. Few experiences regarding pyoderma gangrenosum and cutis laxa are reported. For the first, topical or oral corticosteroids can help, although infliximab has shown good response rates. Treatment of acquired cutis laxa is based on the underlying monoclonal gammopathy (Table 2).

## 4. M-Protein Related Bleeding Disorders

Bleeding disorders in monoclonal gammopathies are related to abnormalities in primary or secondary hemostasis. It is well known that there is a relationship between AL amyloidosis and factor X (FX) deficiency due to the adsorption of FX by amyloid fibrils that decreases its half-life time causing bleeding complications [47]. Acquired von Willebrand disease is another bleeding disorder that results in mucocutaneous bleeding in patients without family history [48]. Laboratory tests show decreased levels of either von Willebrand factor (VWF), ristocetin cofactor, or high molecular weight multimers [49]. There are cases where the underlying monoclonal gammopathy was MGUS, WM, MM, or AL amyloidosis [23,50,51]. For patients who need immediate treatment, desmopressin and factor VIII (FVIII) concentrates can improve symptoms [49]. IVIG is also an option in patients with MGUS [48]. However, definitive treatment depends on the underlying gammopathy.

Platelet aggregation disorders in monoclonal gammopathies have been associated to the presence of a serum M-protein. It has been postulated that the paraprotein binds to platelet receptors involved in aggregation. This leads to prolonged bleeding time and, in some patients, causes unexplained mucocutaneous bleeding or bruising or in others can cause severe bleeding, resulting in hematuria or large hematomas [52,53].

Clinical case 7: A 38-year-old male without prior medical history was admitted because of severe macroscopic hematuria and clots, causing acute kidney injury. During the admission, imaging studies revealed multiple clots along the urinary tract with no other relevant findings. Coagulation tests and platelets count were normal. Serum immunofixation was positive for IgG-lambda of 15.7 g/L. Urine immunofixation was negative, and the 24-hour urine protein excretion did not show proteinuria. The fat biopsy was negative for Congo red staining. The bone marrow showed 11% of plasma cells. It was considered to perform a kidney biopsy but was otherwise normal, and no complement or immunoglobulin deposits were seen in the immunofluorescence. In this scenario, the patient was diagnosed with unknown severe hematuria and a concomitant IgG-lambda smoldering myeloma. The patient was kept under supportive treatment, showing complete resolution of the episode. He was referred to the hematology and nephrology outpatient clinics for follow-up. One and a half year later, the patient was admitted because of recurrent huge iliac psoas hematoma with no previous traumatic injury. The episodes resolved spontaneously, but more tests were performed. The platelet aggregometry assay showed an absence of response to ADP and a decreased liberation with agonists. These results were consistent with a platelet aggregation disorder related to the IgG-lambda M-protein. The patient was started on four cycles of cyclophosphamide, bortezomib, and dexamethasone followed by ASCT. He achieved serological VGPR (IgG-lambda only detectable by immunofixation) with no recurrence of the bleeding symptoms. Four years later, the patient presented again with every transient episode of hematuria and small hematoma in the pelvic area with spontaneous resolution. Serum IgG-lambda M-protein increased up to 12 g/L and lambda serum free light chain of 36 mg/L. He was diagnosed with relapse of the M-protein bleeding disorder. He started treatment again with four cycles of cyclophosphamide, bortezomib, and dexamethasone followed by a second ASCT. He achieved serological VGPR with a stable IgG-lambda M-protein lower than 2 g/L. He is completely asymptomatic now, two years beyond the second ASCT.

*Treatment summary recommendation of M-protein related bleeding disorders*. Whether the bleeding disorder is caused by an acquired von Willebrand syndrome or a platelet aggregation disorder, supportive treatment with coagulation factors is mandatory in case of life-threatening bleeding or given as prophylaxis before procedures. Definitive treatment is against the underlying monoclonal gammopathy, as it can reverse the hemostatic abnormalities. A benefit-to-risk approach should be made in patients with MGUS. However, as the disease consists of bleeding and is potentially life-threatening, anti-myeloma therapy is recommended (Table 3).

## 5. Ocular M-Protein Related Diseases

There are few reports about ocular disorders related to paraproteinemia. Most of them are manifested as keratopathy. Corneal immunoglobulin deposition is described as dot-like crystals or patch-like in the cornea layers. Immunohistochemistry shows light- or heavy-chain immunoglobulin deposits. Photophobia with spared visual acuity is the most frequent symptom [15,54]. However, progressive corneal thickening with central involvement may lead to visual loss.

Asymptomatic patients with corneal deposits related to an MGUS should be closely followed without the need of treatment. In the presence of progression with the risk of visual loss, a bortezomib-based regimen should be initiated. Consolidation with high-dose melphalan followed by ASCT achieves high rates of hematological and clinical response in patients with LCDD [55]. In a study with 169 patients with LCDD and/or HCDD, the overall response rate was 67% (30% with complete response) after ASCT [19]. Risks and benefits should be carefully evaluated when the presentation is atypical (such as clinical case 8) or does not involve kidneys. Importantly, recent studies report that extrarenal involvement can be seen in up to 35% of patients with LCDD or HCDD [19].

**Figure 6:**
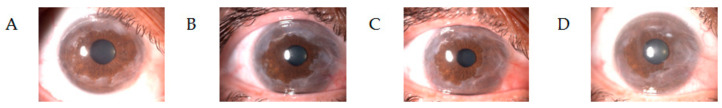
Images of corneal kappa light chain deposition disease. (**A**) Peripheral corneal deposits at diagnosis. (**B**) Three years later, the ocular examination revealed increased corneal involvement. (**C**) Picture taken before the autologous stem cell transplant (ASCT) showing stable disease (**D**) One year after ASCT, the patient achieved stringent complete response with stabilized corneal involvement.

Clinical case 8: A 36-year-old female without other relevant medical history was diagnosed with IgG-kappa MGUS (4% of bone marrow plasma cells, M-protein size of 25 g/L, and normal skeletal survey) during routine work-up tests. She was kept under follow-up at the hematology outpatient clinic. Eight years later, the patient complained of mild photophobia and ocular pain. The ocular examination revealed corneal deposits in both eyes; visual acuity was otherwise normal. The corneal biopsy demonstrated kappa free light chain deposits by immunohistochemistry. No extracorneal involvement was detected. At that time, serum kappa free light chain increased up to 174 mg/L, and tests revealed a small amount of urine M-protein of 279 mg/24 h. The serum M-protein was 27.6 g/L. In this context, the patient was diagnosed with LCDD with mild corneal involvement. As visual acuity was normal, with only peripheral corneal involvement, local treatment was indicated, and she was kept under observation. Three years later, the photophobia increased with ocular pain. At that time, she had a serum M-protein of 22 g/L, serum kappa free light chain increased up to 238 mg/L, serum involved/uninvolved free light chain was 35.7, and the bone marrow aspirate had 4% of plasma cells. Skeletal survey did not show lytic lesions, and fat biopsy was negative for amyloid. Ophthalmological examination revealed increased corneal deposits. She was started on bortezomib and dexamethasone for four cycles and underwent ASCT conditioned with high-dose melphalan. She achieved stringent complete response with negative minimal residual disease and resolution of ocular symptoms with no progression of corneal deposits on the following visits. Figure 6 shows consecutive ocular photographs since diagnosis.

*Treatment summary recommendation of ocular-related disease.* Patients without significant symptoms should be followed with a watch and wait strategy. However, when symptoms worsen with a risk of visual loss, the need of treatment is mandatory. As in other types of LCDD, treatment with anti-myeloma agents can achieve clinical and hematologic responses with long-lasting remissions (Table 3).

## 6. Neurologic M-Protein Diseases

### IgM Peripheral Neuropathy

Peripheral neuropathy is the most frequent neurological syndrome associated with monoclonal gammopathies [56]. By far, the association is stronger and more frequent when the IgM isotype is involved (related to either IgM MGUS or Waldenström macroglobulinemia) [21]. Rarely, IgG or IgA can be attributed as cause of the peripheral neuropathy in a patient otherwise diagnosed with MGUS; however other etiologies should be explored. Indeed, MGUS prevalence increases with age as well as other frequent causes of peripheral neuropathy (i.e., diabetes), raising the possibility of coincidence instead of causality.

IgM gammopathies have usually an underlying pathogenic mechanism that could explain the development of peripheral neuropathy. Patients with IgM MGUS and neuropathy can develop different clinical phenotypes; however, the most frequent one is a distal, symmetric, and demyelinating neuropathy associated with antibodies directed against MAG (myelin-associated glycoprotein). Thus, anti-MAG peripheral neuropathy accounts for about 50% of IgM peripheral neuropathy. This syndrome is usually seen in patients older than 60 years old, with insidious onset and with progressive significant disability. Serum ELISA can show high titers of anti-MAG with a good specificity, but titers are not linked to severity. Electrophysiological studies demonstrate a distinctive pattern with slow conduction and prolonged distal motor and sensory latencies [20,57]. Rituximab has proven to be active in clinical trials [58,59,60], in general showing stabilization of the neurological disease. Other therapeutic options are IVIG or corticosteroids, but stabilization is usually achieved with minor responses [21,61]. A recent, short prospective study based on immunochemotherapy with rituximab, cyclophosphamide, and prednisolone has reported to be effective treating IgM peripheral neuropathy [62]. Ibrutinib is also a promising agent with high rates of response, both hematological and neurological. [63,64].

More rarely, some patients can show anti-ganglioside (GM1) antibodies, since motor neuropathy is the main clinical feature. When no specific autoantibodies can be found on screening tests, IgM MGUS peripheral neuropathy usually presents as a painless chronic distal neuropathy with sensory symptoms and, in some cases, tremor or ataxia. Electrophysiological studies show a demyelization pattern [65,66]. As suggested by the International Workshop on Waldenström Macroglobulinemia (IWWM) 8 consensus, rapid progression should be carefully evaluated and raise the possibility of AL amyloidosis or cryoglobulinemia [21]. If no other cause is established, the presence of a monoclonal IgM in serum could be enough to explain the cause of the peripheral neuropathy [5]. Treatment is recommended for patients with significant disability or progressive symptoms. IVIG, PE, or corticosteroids are first options, while rituximab alone or in combination with alkylating agents can be considered for refractory patients [21,61].

Clinical case 9: A 72-year-old male was referred because worsening of chronic distal symmetrical dysesthesias over the last year. Neurological examination and electrophysiological studies showed findings consistent with a peripheral demyelinating polyneuropathy. Lab tests showed the presence of a serum monoclonal IgM-kappa of 3 g/L without any other abnormality. Anti-MAG antibodies by Dot-Blot were positive, while testing for anti-gangliosides antibodies was negative. Bone marrow aspirate had 10% of normal lymphocytes by morphology. Immunophenotypic analysis showed mature B lymphocytes without kappa or lambda restriction. *MYD88* L265P mutation was negative by AS-PCR. In this context, the patient was diagnosed with anti-MAG peripheral neuropathy related to the IgM MGUS. Given the significant disability, the patients started treatment with four cycles of rituximab 375 mg/m2 weekly. Two months later, the patient had only mild distal sensory symptoms. During the 3-year follow-up, the disease was stabilized with no progression.

*Treatment summary recommendation of neurologic-related disease*. Single-agent rituximab is the first option for patients with anti-MAG IgM peripheral neuropathy or anti-ganglioside antibodies, with ibrutinib being the most promising option in refractory patients. For IgM MGUS peripheral neuropathy without autoantibodies, immunosuppressive treatment may be the first option, while PE, rituximab, immunochemotherapy, or ibrutinib can be considered for unresponsive patients (Table 3).

## 7. Future Directions

Future directions need to be focused on two points. The first one is related to the pathophysiology of the disease: whether there are immune or molecular pathways underlying MGCS that are different from other MGUS and could be related to the clinical features observed. The description of these mechanisms can elucidate new targets and drugs for specific treatment in these diseases. In regard with the latter, a study reported that Schnitzler syndrome has a characteristic activation of the inflammasome compared to healthy controls and raises a question about a distinct mechanism in patients with monoclonal gammopathies [26]. Another recent study reported that TGF-β and collagen 1a mRNA were highly expressed in scleromyxedema skin samples by transcriptomic analysis compared to matched controls [27]. These data not only help to characterize the different types of MGCS but might lead to better targeted therapies for patients. For instance, it was also reported that TGF-β was inhibited after using IVIG in scleromyxedema patients [67]. Regarding anti-MAG neuropathy, the description of its clonal genomic status gives more argument for using Bruton Tyrosine Kinase inhibitors in this group of patients [28]. Another promising strategy for this syndrome is the development of a glycopolymer that mimics the HNK-1 glycoepitope (the anti-MAG antibody target) [22]. Other novel therapeutic options are related to new pathways in MGCS. In this regard, junctional adhesion molecule A (JAM-A), a novel, overexpressed molecule in MM related to angiogenesis, is a potential target [68]. The role of lyso-glucosylceramide (LGL1) to act as an antigen in the monoclonal gammopathy related to Gaucher disease could be another potential target. Moreover, it is reported that reactivity among lysolipids and monoclonal immunoglobulins may trigger the proliferation of aberrant plasma cells in sporadic MGUS [69]. Taken together, deeply understanding of the immune background dysregulation could add more therapeutic options in the future, involving target antigen reduction. 

The second point that remains to be elucidated is how to predict which patients might develop MGCS. We already know that comorbidities not related to progression to symptomatic disease are higher in patients with MGUS [70,71]. We are lacking clinical or laboratory features to identify which patients are at higher risk of MGCS development or a specific test to diagnose these entities, except for anti-MAG antibodies. Moreover, the prognosis of patients with MGCS still remains unknown, in part because of its heterogeneity and rarity with the diagnostic challenges adding more complexity. Few reports attempted to describe the risk of progression from MGCS to symptomatic MM or other lymphoproliferative disorder. For instance, a series with long follow-up reported that 8% of patients with Schnitzler syndrome progressed to a lymphoproliferative disorder [72]. In another series, progression to WM or amyloidosis was observed in 3 out of 22 patients with anti-MAG neuropathy. For other MGCS, studies with short follow-up or smaller samples were not capable of establishing a prognosis. Longitudinal prospective studies of collaborative groups might answer this question. For instance, a nationwide prospective study currently ongoing in Iceland for screening and follow-up of MGUS may give some insights [73].

## 8. Conclusions

MGCS is a newly emergent concept. Screening for an underlying malignancy, such as MM, WM, AL amyloidosis, or other lymphoproliferative disorders, is mandatory. Treatment is based on the presence of symptoms, particularly if they cause disability. When the diagnosis is established, a risk to benefit approach is the first step. Many of these MGCS are diagnosed in the setting of an already established disease. The next approach should be to assess the M-protein isotype involved, as non-IgM-related diseases are treated with anti-myeloma agents, while anti-CD20-based regimens are the preferred option for IgM-related diseases. Although not enough data are available, this review summarizes the treatment possibilities for MGCS (Table 2 and Table 3) and gives insight into new potential therapeutic targets. Both hematological and clinical response should be the main goals after treatment. High-dose melphalan followed by ASCT has to be considered for fit patients. In our experience, this approach is safe and can result in long-term remissions. Finally, we consider that high-throughput technologies analyzing both the plasma/B-cell clones and the bone marrow immune microenvironment might answer unsolved questions in MGCS and find new potential targets.

## Figures and Tables

**Table 1:** Overview of monoclonal gammopathy of clinical significance. M-protein, monoclonal protein; MGUS, monoclonal gammopathy of undetermined significance.

Affected Organ	Disease
Skin	Type 1 cryoglobulinemia Schnitzler syndrome Pyoderma gangrenosum Scleromyxedema Acquired generalized cutis laxa
Neurologic M-protein-related diseases	IgM MGUS neuropathy IgG/IgA MGUS neuropathy
Ocular	Paraproteinemic keratopathy
M-protein-related bleeding disorders	Acquired von Willebrand syndrome Impaired platelet aggregation

**Table 2:** Summary of treatment recommendations for skin conditions in MGCS. M-protein, monoclonal protein; Anti-IL1, anti-interleukin 1; anti-TNF, anti-tumoral necrosis factor; IVIG, intravenous immunoglobulins; anti-myeloma therapy: proteasome inhibitors, immunomodulatory drugs, +/− high-dose melphalan with autologous stem cell transplant.

Disease	Underlying Mechanism	M-Protein Isotype	Treatment
Type 1 cryoglobulinemia	Monoclonal immunoglobulin crystallization. Cold exposure is a trigger to induce aggregation of cryoglobulins (skin) or other unknown factors (kidney, nerves).	IgG, IgM	Glucocorticoids Alkylating agents (i.e., cyclophosphamide) PE Rituximab (IgM type) Anti-myeloma therapy (non-IgM types)
Schnitzler syndrome	Inflammasome upregulation leads to IL-1β and IL-18 release. IgM deposits in the skin of patients with rash (possible autoantibody effect). Suspected genetic predisposition: *NLRP3* mutation.	IgM, (rarely IgG)	Anti-IL1 (anakinra) Oral prednisone Rituximab or ibrutinib Anti-myeloma therapy (non-IgM)
Pyoderma gangrenosum	Interaction between monoclonal IgA with its receptors that leads to cytokine release and pro-inflammatory mediators (IL-6, EGF, MCP-1). Abnormal activation of neutrophils.	IgA, (rarely IgM)	Topical or oral prednisone Anti-TNF (infliximab) Steroid-sparing drugs (cyclosporine A, mycophenolate, tacrolimus) Anti-myeloma therapy if refractoriness
Scleromyxedema	High expression of TGF-β, and collagen-1a might increase proliferation of fibroblasts. Reduced levels of pro-inflammatory mediators are seen after IVIG therapy.	IgG	IVIG for non-severe symptoms Anti-myeloma therapy for refractory or severe symptoms
Acquired cutis laxa	Elastic fiber destruction by phagocytosis after monoclonal immunoglobulin deposition Elastic fiber destruction mediated by complement.	IgG	Anti-myeloma therapy

**Table 3:** Summary of treatment recommendations for other infrequent MGCS. IVIG, intravenous immunoglobulins; anti-MAG, anti-myelin associated glycoprotein; anti-myeloma agents: proteasome inhibitors, immunomodulatory drugs, +/− high-dose melphalan with autologous stem cell transplant; VWF, von Willebrand factor; HNK-1, human natural killer-1.

Disease	Underlying Mechanism	M-Protein Isotype	Treatment
Platelet aggregation disorder	Aberrant deposition of monoclonal immunoglobulin on platelet surface targets (glycoprotein IIIa, GP1b).Immunologic destruction of VWF (autoantibody activity).	IgG	Anti-myeloma therapy
Keratopathy	Crystalline monoclonal immunoglobulin deposits or non-organized light-chains deposits on corneal surface. Overproduction of abnormal immunoglobulin conformation, impaired enzymatic degradation, and high tropism for organ deposition.	Heavy or light chains	Anti-myeloma therapy
Peripheral neuropathy	Monoclonal IgM targets HNK-1 epitope on MAG glycoprotein causing demyelinating lesions (autoantibody activity). Other potential targets: gangliosides (GM1, GM2, GM3, GD1a, GD1b, GT1b), and paraglobosides.	IgM	Anti-MAG/ganglioside: Rituximab No antibodies or non-IgM neuropathy: IVIG, prednisone, anti-myeloma agents

## Data Availability

The data presented in this study are available in this article (see References) and on request from the corresponding author.
